# The novel pleuromutilin derivative 22–((4-((4-nitrophenyl)acetamido)phenyl)thio)deoxy pleuromutilin possesses robust anti-mycoplasma activity both *in vitro* and *in vivo*

**DOI:** 10.3389/fphar.2024.1491223

**Published:** 2024-12-20

**Authors:** Xirui Xia, Xuan Ji, Yaxi Li, Yubo Wang, Yue Zhao, Wenxiang Wang, Huanzhong Ding

**Affiliations:** Guangdong Key Laboratory for Veterinary Drug Development and Safety Evaluation, College of Veterinary Medicine, South China Agricultural University, Guangzhou, China

**Keywords:** 22-((4-((4-nitrophenyl)acetamido)phenyl)thio)deoxypleuromutilin, pharmacokinetic, pharmacodynamic, *Mycoplasma pneumoniae*, antibacterial activity

## Abstract

**Objective:**

Mycoplasmas are structurally simple pathogenic microorganisms that can cause a wide range of diseases in humans and animals and conventional antibiotic therapies of fluoroquinolones and tetracyclines are toxic to young children and young animals and macrolide resistance is increasing. In this context, new anti-mycoplasma antimicrobial agents need to be developed. 22–((4-((4-nitrophenyl)acetamido)phenyl)thio)deoxypleuromutilin (compound 16C) is a novel acetamine phenyl pleuromutilin derivative. This study aimed to evaluate its acute toxicity in mice and generate pharmacokinetic and anti-mycoplasma profiles.

**Methods:**

The safety of compound 16C was preliminarily evaluated by oral and intramuscular acute toxicity tests and single intravenous and intramuscular pharmacokinetic experiments were performed to obtain its pharmacokinetic profile. The minimum inhibitory concentration (MIC), minimum bactericidal concentration (MBC), and time-killing curves reflected the *in vitro* effects of the compounds against *Mycoplasma pneumoniae.* Five groups consisted of three treatments for compound 16C (20, 40, and 80 mg/kg), and two treatments for tiamulin (oral and intramuscular 40 mg/kg) were continued for 4 d. Bronchoalveolar lavage fluid (BALF) and lung tissues were collected at the end of treatment (96 h) and 4 days later (192 h) to assess the *in vivo* anti-mycoplasma and anti-pneumonia effects. ELISA assays were performed to detect IFN-γ, TNF-α, and IL-8 (CXCL1) in BALF. Lung tissues were fixed with 4% paraformaldehyde and sectioned for histopathological assessment.

**Results:**

The results show that compound 16C has low toxicity (LD_50_ > 5,000 mg/kg). Its pharmacokinetic profile is characterized by a short time to maximum concentration (Tmax = 0.24 h), high bioavailability (F = 71.29%), and short elimination half-life (T_1/2kel_) (intramuscular and intravenous administration was 2.20 and 1.89 h, respectively). Treatment with compound 16C and intramuscular tiamulin reduced the *mycoplasma* load in mice. Intramuscular compound 16C and tiamulin also inhibited the release of IFN-γ, TNF-α, and CXCL1, decreasing the accumulation of inflammatory cells in the lungs, thereby mitigating lung damage.

**Conclusion:**

This study proved that compound 16C has a strong antimicrobial effect against *M. pneumoniae*, can be rapidly absorbed and has therapeutic efficacy that provides a basis for developing new anti-mycoplasma drugs.

## Introduction

Mycoplasmas are small (0.1–0.3 µm), mostly extracellular parasites that possess the most compact genomes (580–2,200 kb) in the bacterial kingdom. This reduced coding capacity has evolved a reliance on host metabolic functions for nutrient acquisition. Mycoplasmas lack cell walls but possess high levels of membrane lipoproteins that directly interact and anchor these organisms to the host cell. The lipoproteins are also antigenic and drive inflammation via pattern recognition receptors in the Toll-like Receptor (TLR) family (Waites and Talkington, 2004; You et al., 2006; Zuo et al., 2009).

Mycoplasmas as infectious agents cause respiratory, genitourinary, and joint diseases in humans and animals (Gnanadurai and Fifer, 2020; Levisohn and Kleven, 2000; Maes et al., 2008). *Mycoplasma pneumoniae* is the primary pathogen of community-acquired pneumonia (CAP). Interestingly, infections in infants are mostly asymptomatic, and pneumonic disease develops in children >10 years old and is a sign of a growing host immune response to the pathogen (Fernald et al., 1975). A cough is the most common clinical symptom of *M. pneumoniae* respiratory tract infection (Gadsby et al., 2012; Maheshwari et al., 2011; Waites and Talkington, 2004). In addition, *M. pneumoniae* and its specific antibodies (IgG) are often detected in the airways of asthmatics and infections can exacerbate airway inflammation (Jiang et al., 2015; Kumar et al., 2019). Bronchial hyperresponsiveness was also noted in a mouse model of *M. pneumoniae* infection following allergic asthma (Chu et al., 2003; Collins et al., 2021; Iannuzo et al., 2022).

Due to the lack of a cell wall, mycoplasmas are intrinsically resistant to antibiotics targeting the cell wall, such as β-lactams, but can be treated with tetracyclines and fluoroquinolones, although undesirable adverse effects include enamel hypoplasia, depression of bone growth, and cartilage hypoplasia in children as well as young animals (Cai et al., 2024; Tsai et al., 2021). Therefore, the most common treatment for *mycoplasma* infections has been the macrolides. However, their widespread use has increased resistance and pediatric CAP cases are becoming increasingly difficult to treat due to macrolide-resistant *mycoplasma* (Ding et al., 2024; Lee et al., 2017; Wang Y. et al., 2024). Therefore, safe and effective treatments that do not contribute to resistance are needed. The therapeutic effects of a series of plant extracts on mycoplasma-induced pneumonia have been investigated in mouse models with a special emphasis on their antioxidant and anti-inflammatory effects. However, the effects of these compounds against *M. pneumoniae* were not reported in detail (Chen et al., 2022; Ding et al., 2023; Fu et al., 2022). The ideal drug candidate would minimize adverse inflammatory effects while still possessing potent bactericidal effects.

Pleuromutilins are terpene derivatives and are secondary metabolites produced by fungi via the mevalonate pathway. These compounds have antimicrobial mechanisms similar to macrolides and interfere with protein synthesis by binding to the peptidyl transferase center of the 23S ribosomal RNA. Tests using clinical *mycoplasma* isolates of veterinary origin have indicated high sensitivity to tiamulin and valnemulin and associated minimal inhibitory concentrations (MIC) were homogeneous and clinical strains of *Mycoplasma synoviae* isolated in Thailand exhibited high susceptibility to tiamulin but developed a tilmicosin-resistant subpopulation, indicating a robust mode of action for the pleuromutilins (Gautier-Bouchardon, 2018; Limpavithayakul et al., 2023). Substituents of pleuromutilin derivatives have also been verified for their influence on drug properties, and the carbonyl group on the cyclopentane ring, as well as the C11 hydroxyl, are necessary to maintain the antibacterial activity of the compounds. In contrast, chemical modifications at the C14 side chain improve their antimicrobial activity and solubility in water (Egger and Reinshagen, 1976). C14 side chains containing a thioether substituent greatly increase the activity of pleuromutilin derivatives, whereas carboxyl groups decrease the activity. The thioether derivatives have more potent antibacterial activity against Gram-positive bacteria than sulfoxide-type derivatives (Hunt, 2000; Li et al., 2019; Raoni Schroeder Borges Goncalves and Vinicius Nora de Souza, 2010). On this basis, a series of compounds containing a thioether substituent were designed and synthesized, and these include tiamulin and valnemulin, which were approved for veterinary use in 1979 and 1999 (Hannan et al., 1997; Hunt and E., 2000; Schlünzen et al., 2004). A series of pleuromutilin derivatives, such as azamulin, retapamulin, and lefamulin, have been developed for human use, and lefamulin is the first pleuromutilin derivative approved for treating systemic infections in humans (Eraikhuemen et al., 2021; Ling et al., 2014; Yan et al., 2006).

A series of derivatives containing 2-aminothiophenol or 4-aminothiophenol on the C14 side chain were previously synthesized in our laboratory. These derivatives displayed better anti-MRSA and *S. aureus* activities and lower cytotoxicity than tiamulin (Chai et al., 2022; Jin et al., 2019; Zhang et al., 2018). Our preliminary screening of these compounds revealed that 22–((4-((4-nitrophenyl)acetamido)phenyl) thio)deoxy pleuromutilin (compound 16C) had good anti-mycoplasma activity, and this prompted us further to investigate the *in vitro* and *in vivo mycoplasma* effects. These results indicated that the MICs of compound 16C were lower than those of tiamulin, vancomycin, valnemulin, and retapamulin for MRSA and *Staphylococcus aureus* (Chai et al., 2022).

In the current study, we evaluated the anti-mycoplasma activities of compound 16C *in vitro* and *in vivo* using pharmacokinetic and acute toxicity assays, time-kill curves and *in vivo* pharmacodynamics in mycoplasma-infected mice.

## Materials and methods

### Chemicals

22–((4-((4-nitrophenyl)acetamido)phenyl)thio)deoxypleuromutilin (compound 16C) was obtained from Chai et al. and stored at −80°C, synthesized using previous methods (Chai et al., 2022). Briefly, pleuromutilin was dissolved in acetonitrile, and *p*-toluenesulfonyl chloride and NaOH were added. The mixture reacted at 0°C for 0.5 h and then at room temperature for 3 h to obtain compound 7. It was purified and dissolved in ethyl acetate, to which 4-aminobenzenethiol was added. NaOH was dissolved in water, and the solution was slowly dripped under an ice bath. The reaction was heated and refluxed at 70°C for 3 h to obtain intermediate compound 8. Compound 8 was purified and dissolved in methanol, then *p*-nitrobenzoyl chloride and K_2_CO_3_ were added, and the reaction was carried out at room temperature for 2 h to obtain the final product of pure compound 16C. The product was purified by silica column chromatography, and its structure was confirmed by ^1^H NMR, ^13^C NMR, and high-resolution mass spectral (HR-MS) analysis. Tiamulin fumarate soluble powder (45%) was purchased from Guangdong Dahuanong Animal Health Products (Guangdong, China). Tiamulin (98%), ciprofloxacin, erythromycin, tetracycline, and other liquid reagents, including acetonitrile and other solvents, were purchased from Macklin Biochemical Technology (Shanghai, China).

### Microorganisms and culture conditions

*Mycoplasma pneumoniae* strain M129 (ATCC 29342) was obtained from American Type Culture Collection (Manassas, VA, United States) and cultured at 37°C in the presence of 5% CO_2_ in the modified SP-4 liquid medium (pH 7.4) containing 2.1% (w/v) CM1166 *mycoplasma* broth base medium (Oxoid, Hants, United Kingdom), 20% (v/v) fetal bovine serum (FBS, Bioexplorer, CO, United States), 0.4% yeast extract (Oxoid), 0.55% (w/v) CMRL 1066 w/L-glutamine culture media powder (MyBioSource, San Diego, CA, United States), 1% D-glucose (Macklin) and 0.004% phenol red (Macklin). The change from red to yellow color of the culture after about 96 h indicated that the *M. pneumoniae* has reached the end of the logarithmic phase of its growth. The solid medium is based on the above formula with the addition of 1% agar, and the characteristic fried egg-like colonies were used for experiments following incubation for 7 d on plates.

### Animals

Equal numbers of male and female adult Specific Pathogen Free (SPF) BALB/c and ICR mice (20–22 g) were purchased from Zhuhai Bestest Bio-Tech (Guangdong, China) and kept in the animal biosafety level 2 facilities at the Laboratory Animal Center of South China Agricultural University. The experimental procedures were performed in accordance with the Ethical Principles in Animal Research and were approved by the Committee for Ethics in Laboratory Animal Center of South China Agricultural University (number: 2023c094).

### Acute toxicity study

#### Pre-test

Twenty four male and female SPF-grade ICR strain mice were randomly divided into 6 groups of 4 mice each. Compound 16C was dissolved in DMSO at doses of 0, 8, 40, 200, 1,000, and 5,000 mg/kg and was administered to the mice by gavage. The mice were fed and watered *ad libitum* and continuously observed for 14 d and no deaths were observed.

#### Formal test

Ten mice were gavaged with 5,000 mg/kg of compound 16C and another 10 mice were given DMSO orally as a control. The mice were fed and watered *ad libitum*. The animals’ appearance, diet, behavior, and death were observed and recorded for 14 consecutive days after administration. The LD_50_ was calculated as previously described (Xiao et al., 2019), and the test was repeated twice. To assess the acute toxicity of intramuscular administration in mice, we repeated the above experiment and replaced gavage with intramuscular injection.

### Hemolysis tests

Hemolysis tests were performed according to previously reported standard methods with appropriate adjustments (Sæbø et al., 2023). Briefly, high-dose compound 16C injection (8,000 μg/mL) and blank formulation were serially diluted 2-fold with PBS (pH 7) 10 × as compound 16C injection group and blank formulation group, respectively. PBS and erythrocyte lysis buffer (Solarbio, Beijing, China) were used as negative and positive controls, respectively. An equal volume of 2% mouse erythrocyte suspension was added to the above solution, mixed upside down and incubated at 37°C for 1 h. After centrifugation at 1700 *g* for 5 min, 100 µL of the supernatant was transferred to a 96-well plate and the absorbance was measured at 405 nm. The tests were repeated 3× with 2 parallels each time.

### Pharmacokinetic studies in mice

Pharmacokinetic experimental procedures of compound 16C were measured according to previous reports with some modifications (Wang et al., 2022). In brief, BALB/c mice were divided into two groups of eight mice each; one group was injected intravenously and the other was injected intramuscularly with compound 16C at a dose of 10 mg/kg. The dose was based on previous studies of novel pleuromutilin derivatives (Jin et al., 2019; Wang et al., 2022). Meanwhile, the dose of tiamulin approved by the European Union for intramuscular administration is 10–20 mg/kg (EMA, 1999). The volume of the injection was 0.1 mL/10 g. The compound 16C formulation was a filtered solution in 5% DMSO, 5% Tween-80, and 90% normal saline. Blood was obtained from eight mice via the ocular venous plexus at 0.083, 0.167, 0.25, 0.334, 0.5, 0.75, 1, 1.5, 2, 3, 4, 6, 8, and 12 h following tail intravenous or thigh Intramuscular injection. A glass capillary with a diameter of 0.3 mm was inserted into the outer canthus of the mouse, and 200 μL blood was collected using a 1.5 mL anticoagulant centrifuge tube. The blood was centrifuged at 3,000 rpm for 10 min to obtain plasma. 100 μL of plasma was aspirated for drug concentration detection, and eight parallel samples from different mice were obtained at each time point.

### Method validation

The intra- and inter-batch variations and extraction recoveries were determined by repeated analysis of compound 16C at three concentration levels (10, 100, and 400 ng/mL). Six plasma samples containing the corresponding drug concentration and one standard sample were prepared for each concentration, and the samples were extracted and assayed. The experiment was repeated three times on three different days. The linearity of the method was determined by analyzing the calibration standard samples (Plasma containing a given concentration of compound 16C) ranging from 1 to 400 ng/mL (1, 10, 50, 100, 200, 400 ng/mL) and the calibration curves were constructed by plotting peak area (y) of compound 16C versus the nominal concentration (x). Plasma samples containing 0.5, 1, and 10 ng/mL drug were prepared, and the actual concentrations were measured. When the signal-to-noise ratios (SNR) of the sample were >10 and 3, the corresponding concentrations were defined as the limit of quantitation (LOQ) and detection (LOD), respectively.

### Extraction method

All pharmacokinetic experimental samples and method validation samples were added with 4 times the volume of acetonitrile and shaken for 1 min, centrifuged at high speed at 13,000× g for 10 min, and the supernatants were taken and filtered to detect the drug concentration. Some samples with high concentrations were diluted to the linear range prior to analysis.

### Sample testing method

The extracted samples were analyzed using a high-performance liquid chromatography-tandem mass spectrometry (HPLC-MS/MS) system (Agilent, Santa Clara, CA, United States) 1200 HPLC and Applied Biosystems (Waltham, MA, United States) API 4000 mass spectrometer). HPLC-MS/MS analysis using a Zorbax Eclipse XDB-C18 (4.6 × 50 mm, 3.5 μm) column (Agilent). The mobile phase A (acetonitrile) and B (water) were applied as a gradient as follows: 0–3 min: 15%–95% A and maintain from 3 min to 5 min at 95% A. 5–5.1 min: 95%–15% A and maintain from 5.1 to 10 min at 15% A. Flow rate: 500 μL/min; column temperature: 30°C and injection volume: 4 μL. The electrospray ionization (ESI) source of the mass spectrometer was operated in negative ion mode, with the following ion source parameters: ion spray voltage 4,500 V, collision gas 7 psi, ion source gas 1 55 psi, ion source gas 2 40 psi, curtain gas 20 psi, source temperature 600°C. The precursor ion m/z of Compound 16C was 633.3, and the product ions m/z were 272.0 (quantifier) 286.9 and 313.0. Declustering potentials (DP) were 140, 140, and 144 V, respectively; entrance potential (EP) was 10 V; collision energy (CE) was 55, 46, and 15 V, respectively; and collision cell exit potential (CXP) was 14 V.

### Pharmacokinetic data analysis

The pharmacokinetic profiles of compound 16C in plasma were analyzed using a two-compartmental model provided in WinNonlin software, Version 5.2.1 (Certara, Princeton, NJ, United States). Pharmacokinetic parameters include time to maximum concentration (Tmax), maximum concentration (Cmax), the area under the plasma concentration–time curve (AUC_0-∞_), clearance rate (CL), the apparent volume of distribution (Vd), bioavailability (F), elimination half-life (T_1/2kel_) and absorption half-life (T_1/2ka_).

### Susceptibility assay

The broth microdilution MIC procedure was performed according to standardized methods for microdilution susceptibility testing of *mycoplasma* species (Waites et al., 2012). The MICs of the compound 16C, tiamulin, ciprofloxacin, tetracycline, and erythromycin against *M. pneumoniae* were determined. The concentration of organisms should be determined to approximately 10^6^ CFU/mL prior to inoculation into 96-well plates. Due to the action of phenol red, the acidic metabolites of microbial growth can turn the medium yellow. When the positive control wells changed from red to yellow (≈96 h), the lowest drug concentration whose color matched the negative control was taken as the MIC result (See supplementary material). The MBC assays were performed based on MICs following endpoint reading and the liquid in the high-concentration wells was removed for bacterial counting. Minimum bactericidal concentration (MBC) was defined as the lowest antibiotic concentration corresponding to wells with <0.1% bacterial inoculum (Pankey and Sabath, 2004).

### Time-kill curves

Based on previous studies, we used a modified *mycoplasma* time-kill curves method (Wang W. et al., 2024) with 0.5 mL strain M129, 0.1 mL test antibiotics (compound 16C, tiamulin, ciprofloxacin, tetracycline, and erythromycin), and 4.4 mL of blank broth added to 10 mL tubes. It resulted in a final microbiological concentration of ∼ 10^7^ CFU/mL and antibiotic concentrations of 0–64 × MIC. The tubes were incubated at 37°C in 5% CO_2_ for 72 h, and 200 µL of the culture was removed every 12 h for 10-fold serial dilutions. The diluted cultures were plated on plates for further culture and counted after 7 d. The experiment was repeated three times.

### *In vivo* pharmacodynamic studies

Adult BALB/c mice (20–22 g) were divided into a control group, a positive group, and compound 16C groups at low (20 mg/kg), medium (40 mg/kg), and high-dose (80 mg/kg) levels and oral and intramuscular tiamulin treatment groups (40 mg/kg). We formulated the above dose range based on the dose at which tiamulin was approved for injection (10–20 mg/kg) and the dose previously reported (45 mg/kg) (EMA, 1999; Xiao et al., 2016). In summary, we selected similar doses for compound 16C *in vivo* study. 40 mg/kg was used as an intermediate value, one-half of it as a low dose, and two times of it as a high dose. To prepare the challenge solution, logarithmic phase culture suspensions were centrifuged at 1700 *g* for 10 min, and the pellets were suspended in sterile PBS to obtain 1 × 10^9^ CFU/mL. Before the challenge, mice were anesthetized with tribromoethanol (250 mg/kg, IP). Mice in the control groups were intratracheally inoculated with sterile PBS, whereas mice in the other groups were intratracheally challenged with 50 μL (1 × 10^9^ CFU/mL) *M. pneumoniae* suspension once a day for 3 days. Compound 16C and tiamulin (tiamulin-IM group) dissolved using the method mentioned in pharmacokinetic studies, and the tiamulin fumarate soluble powder was dissolved in ddH_2_O for oral administration. After the final challenge, antibiotics were administered at low, medium, and high-dose levels (see above) at 10 a.m. and 10 p.m. daily for 4 consecutive days. Normal saline containing 5% DMSO and 5% Tween-80 was administered as control and positive groups by thigh intramuscular injection. To compare the efficacy of compound 16C and tiamulin under the same route of administration, we used the original tiamulin for intramuscular administration in the same formulation. On the other hand, tiamulin fumarate soluble powder, approved for oral administration to treat animal *mycoplasma* diseases in China, was administered to mice by gavage. Based on a clinical study of lefamulin in community-acquired bacterial pneumonia (CABP), subjects were administered twice a day and the 96 ± 24 h of starting treatment was an essential point for early clinical response (ECR) (Paukner et al., 2022). We therefore stopped treatment at 96 h and sampled at 192 h to explore whether the disease worsened after cessation of treatment. Bronchoalveolar lavage fluid (BALF) and lungs were collected at 0, 96, 192 h (Samples of 0 h were collected before starting treatment, and mice were fed *ad libitum* without drug treatment during 96–192 h) after the first dose, BALF for cytokine and cytological analysis and lung for histopathological evaluation.

### Analysis of bronchoalveolar lavage fluid (BALF)

Bronchoalveolar lavage was performed according to a standardized method (Van Hoecke et al., 2017). BALF was obtained following infusion of 0.5 mL PBS via tracheal cannulation into the lung twice (total 1 mL), and 200 µL was used for *mycoplasma* counts. The remaining BALF was centrifuged at 300 *g* for 10 min to separate the debris. The supernatants were stored at −80°C, and the debris was resuspended in the same volume of PBS containing red blood cell lysis buffer. A hemocytometer was used to count total cell numbers in BALF, and cell smears (Diff-Quik staining) were prepared to calculate the percentages of neutrophils, macrophages, and lymphocytes. IFN-γ, TNF-α, and CXCL1 levels in BALF samples were assessed using commercial ELISA kits using guidelines recommended by the manufacturer (Elabscience, Wuhan, China). Parallel data for each experimental group at each time point were obtained from six different mice.

### Histopathological assessment

Lung tissues were collected at 0, 96, and 192 h following drug administration. Lungs were collected from 6 mice in each group at 96 h and 13–15 mice in each group at 192 h. In addition, the lungs of 8 untreated infected mice were taken at 0 h as initial controls. Lung tissues were fixed in 4% paraformaldehyde, and whole-mount sections of paraffin-embedded lungs were stained with hematoxylin and eosin (H&E). The histopathologic score (HPS) was determined according to published protocols by a single pathologist who was unaware of the treatment status and groups of the animals (Cimolai et al., 1992). The HPS was based on individual grading of peribronchiolar/bronchial infiltrate, bronchiolar/bronchial luminal exudate, perivascular infiltrate, and parenchymal pneumoniae. According to the histopathologic score of lung tissue sections at 192 h, the response rate and recovery rate of treatment were calculated.

### Statistical analysis

Prism 8.4.0 software (GraphPad, Boston, MA, United States) was used for statistical analysis, and one-way-ANOVA and all data are presented as mean ± SD. P values reflect the difference between the data in each group. P< 0.05, the difference was significant; P< 0.01, the difference was extremely significant.

## Results

### Acute toxicity of compound 16C

Our initial investigations were to determine whether compound 16C possessed overt lethality in mice. Administration of the drug by either oral gavage or intramuscular injection with a single dose of 5,000 mg/kg did not result in any mouse deaths after 2 weeks. However, we noticed anorexic symptoms in the mice, but the mice rapidly recovered and appeared similar to the control mice. We calculated that the LD_50_ of compound 16C was >5,000 mg/kg.

### Hemolysis tests

**FIGURE 1:**
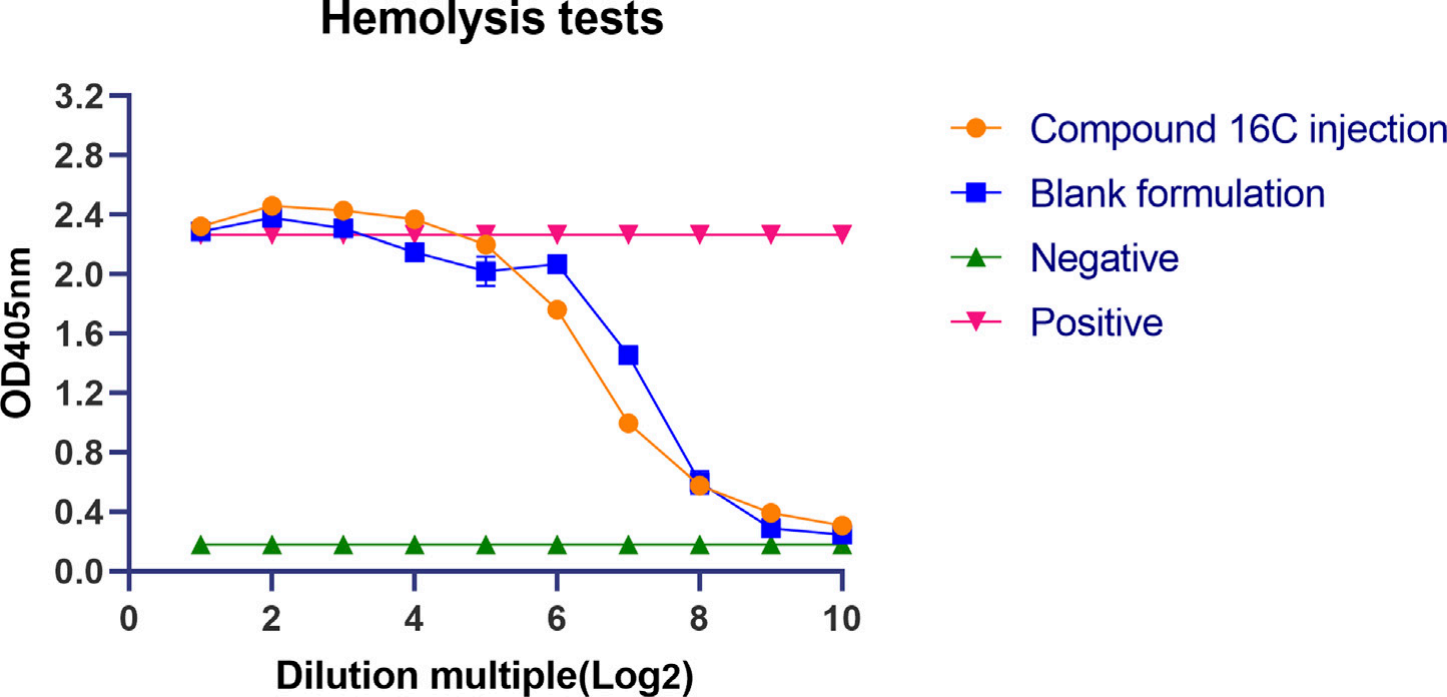
Lysis of mouse erythrocytes by compound 16C injection and its solvent. Orange circles represent Compound 16C Injection; blue squares represent blank formulations; green positive triangles represent negative controls; red inverted triangles represent positive controls.

Compound 16C injection and the blank formulation showed high hemolytic effects at high concentrations with OD_405nm_ values similar to the positive controls (OD_405nm_ = 2.27 ± 0.02), which decreased significantly after the seven serial dilutions. At that time, the antimicrobial agent concentration was 62.5 μg/mL in 0.04% DMSO and Tween 80. After continued dilution, the OD405 nm for both groups decreased to the level of negative control (OD_405nm_ = 0.18 ± 0.0026) (Figure 1).

### Pharmacokinetic analysis

**FIGURE 2:**
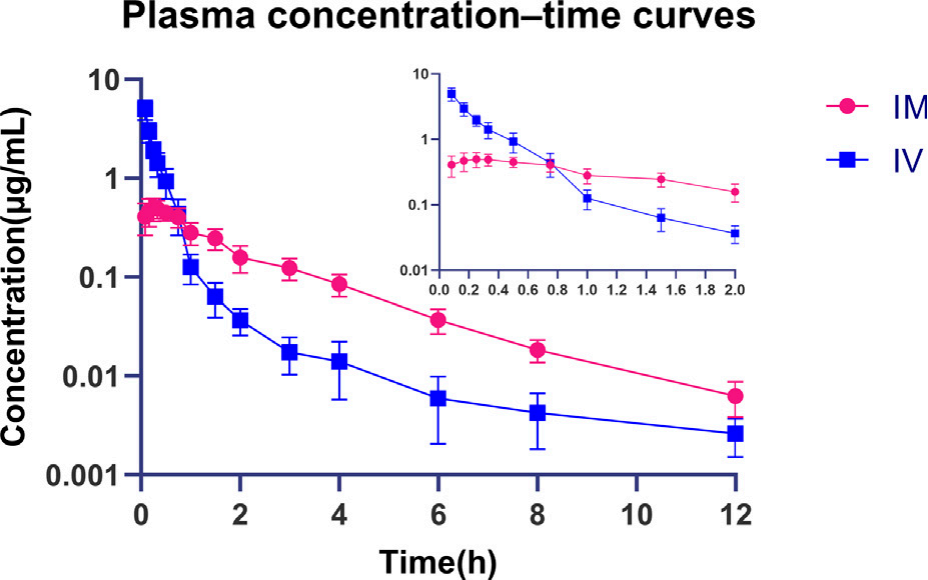
Plasma concentration-time curves of compound 16C during intravenous and intramuscular administration in mice. Blue squares represent intravenous administration; Red dots represent intramuscular administration. n = 8 at each time point.

We performed pharmacokinetic experiments, and the method was initially validated, and compound 16C recovery from plasma extractions ranged from 93.62% to 104.46% with intra- and inter-assay CVs of 4.66%–8.94% and 3.60%–5.27%, respectively. Good linearity was observed for concentrations between 1 and 400 ng/mL (*r* ^2^ > 0.99). The LOD and LOQ were 0.5 and 1 ng/mL, respectively. The pharmacokinetics of compound 16C in mice were studied using intravenous bolus and intramuscular injections at 10 mg/kg (concentration-time curves). The pharmacokinetic parameters were calculated based on a two-compartmental model. Compound 16C was absorbed rapidly, the half-life of absorption (T_1/2ka_) was 0.06 h, and plasma maximum concentration (Cmax) was 0.50 μg/mL after intramuscular administration of the drug into mice at 0.24 h (Tmax). An intravenous bolus generated initial high blood concentrations (C0 = 5.58 μg/mL) and then decreased rapidly. The elimination half-lives (T_1/2kel_) of compound 16C were 2.20 and 1.89 h via intramuscular and intravenous administration, respectively. The areas under the plasma concentration-time curve (AUC_0-∞_) for intramuscular and intravenous administration within 0-∞ were 1.07 and 1.56 h μg/mL, respectively. The clearance rate (CL) was 6.73 L/h/kg, and the apparent volume of distribution (Vd) was 3.55 L/kg. The bioavailability (F) of compound 16C from the intramuscular route was 71.29% (Figure 2; Table 1).

**TABLE 1:** Pharmacokinetic parameters of compound 16C in mice (n = 8).

Parameter	IM (10 mg/kg)	IV (10 mg/kg)
Cmax (µg/mL)	0.50 ± 0.11	—
C0 (µg/mL)	—	5.58 ± 1.25
Tmax (h)	0.24 ± 0.09	—
Ka (1/h)	14.41 ± 7.76	—
Kel (1/h)	0.33 ± 0.07	0.41 ± 0.14
T_1/2ka_ (h)	0.06 ± 0.03	—
T_1/2kel_ (h)	2.20 ± 0.52	1.89 ± 0.61
AUC_0-∞_ (h·µg/mL)	1.07 ± 0.23	1.56 ± 0.37
Cl (L/h/kg)	—	6.73 ± 1.67
Vd (L/kg)	—	3.55 ± 1.32
F (%)	71.29 ± 19.34	—

### Susceptibility assays

Our test antibiotics’ MIC and MBC values against *M. pneumoniae* strain M129 were examined to begin an initial assessment of antibiotic effectiveness. The respective MIC and MBC values were as follows: compound 16C (0.00195312, 0.0078125 μg/mL), tiamulin (0.0078125, 0.0625 μg/mL), erythromycin (0.016, 0.032 μg/mL), tetracycline (0.4, 3.2 μg/mL) and ciprofloxacin (4, 8 μg/mL). The MBC/MIC ratios of these compounds were all ≤4, indicating good *in vitro* bactericidal activities (Zhang et al., 2020).

### Time-kill curves

**FIGURE 3:**
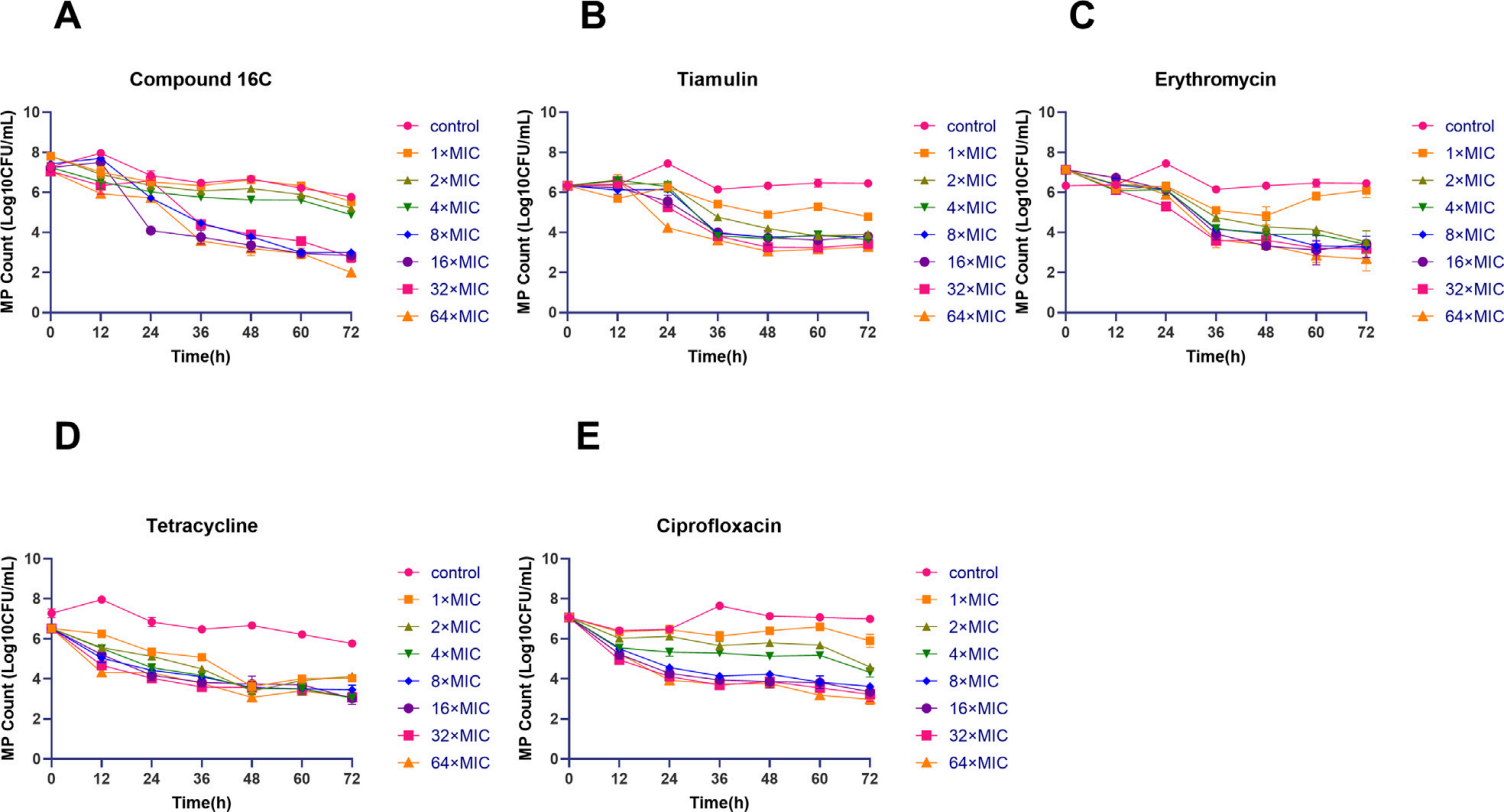
Time-kill curves of the indicated antimicrobial agents against *Mycoplasma pneumoniae* strain M129 (ATCC 29342). Letters represent the *in vitro* antibacterial curves. Time-kill curves of compound 16C **(A)**; time-kill curves of tiamulin **(B)**; time-kill curves of erythromycin **(C)**; time-kill curves of tetracycline **(D)**; time-kill curves of ciprofloxacin **(E)**. Different shapes and colors indicate the control group (without antibiotics) and the antibiotic groups containing 1–64× MIC antibiotics. The data are from three repetitive experiments, and the error bars represent the SD values.

Static time-kill curves were also developed at 0–64× MIC for each drug, and by 72 h, the drugs could be differentiated based on their antimicrobial effects. Compound 16C was bacteriostatic at 1–4 × MIC (2.27–2.60 log_10_ CFU/mL reductions) and was bactericidal at ≥ 8×MIC and reduced bacterial counts >3 log_10_ CFU/mL. Additionally, exposure to compound 16C for 72 h at 64 × MIC decreased the number of organisms to the LOD (2 log_10_ CFU/mL) (Figure 3A). In contrast, tiamulin did not achieve bactericidal effect except at 64 × MIC (Figure 3B). However, erythromycin displayed excellent bactericidal activity at 2 × MIC. Following 72 h exposure, the bacterial load had decreased by 3.61 log_10_ CFU/mL (Figure 3C). Tetracycline inhibited *M. pneumoniae* growth. It achieved 2.38–2.48 log_10_ CFU/mL reductions at 1–2×MIC and was bactericidal at ≥ 4×MIC (Figure 3D). A bactericidal effect was achieved using ciprofloxacin at ≥ 8× MIC causing 3.44 log_10_ CFU/mL reductions in organism density (Figure 3E).

### *In vivo* antibacterial activity

**FIGURE 4:**
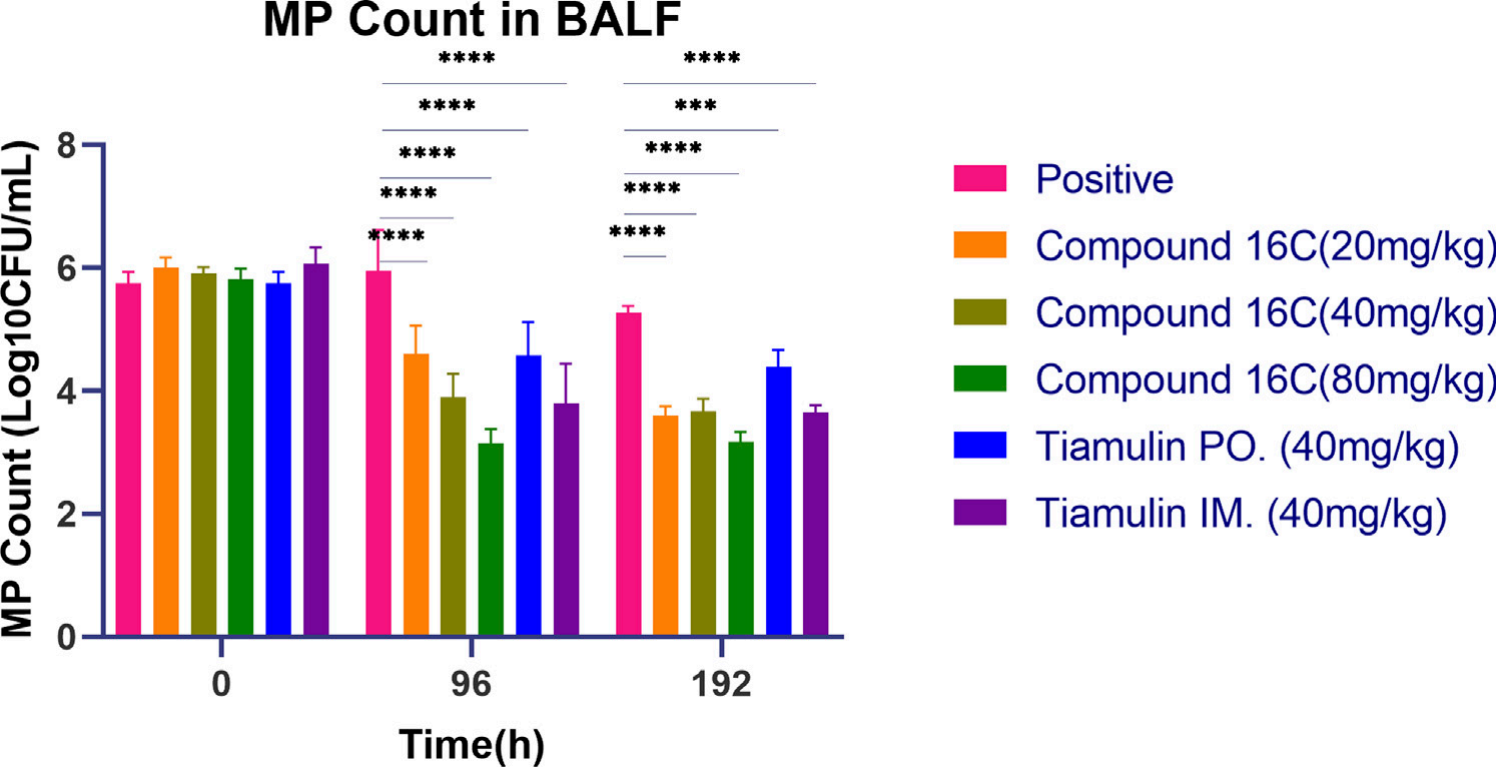
*Mycoplasma pneumoniae* loads in BALF of mice treated with three dosages of compound 16C and tiamulin (PO. and IM.) as indicated and positive control groups. *P< 0.05; **P< 0.01; ***P< 0.001; ****P< 0.0001. n = 6 at each time point.

The *in vivo* antimicrobial activities (Figure 4) of these antibiotics indicated no significant (P> 0.05, n = 6) differences in the initial bacterial load for these six groups in the mouse model of infection. In contrast, by 96 h, the bacterial counts of each treatment group were significantly lower than that of the positive control group. In particular, high-dose compound 16C displayed the most robust ability to reduce bacterial loads (80 mg/kg, IM, microbial reduction, 2.68 log_10_ CFU/mL) (P< 0.0001, n = 6). In comparison, a medium dose of tiamulin orally administrated (40 mg/kg, PO, microbial reduction: 1.17 log_10_ CFU/mL) was similar to low dose compound 16C (20 mg/kg, IM, microbial reduction: 1.40 log_10_ CFU/mL) (P> 0.05, n = 6). A medium dose of tiamulin was administered IM at 40 mg/kg and gave a reduction in bacterial growth of 2.26 log_10_ CFU/mL that was similar to IM compound 16C at 40 mg/kg that gave a reduction of 2.02 log_10_ CFU/mL (P> 0.05, n = 6). Four days after the cessation of treatment, the number of bacteria in each treatment group remained low. It did not recover, indicating that compound 16C and tiamulin were both effective in inhibiting the growth of *M. pneumoniae* in the mouse respiratory system. Meanwhile, the difference in bacterial load between the oral tiamulin group and the other groups was significant (P< 0.01). These results also indicated that compound 16C possessed good *in vivo* antibacterial activities, and the i.m. mode of administration would facilitate treatment protocols.

### Inflammatory cell analysis in BALF

**FIGURE 5:**
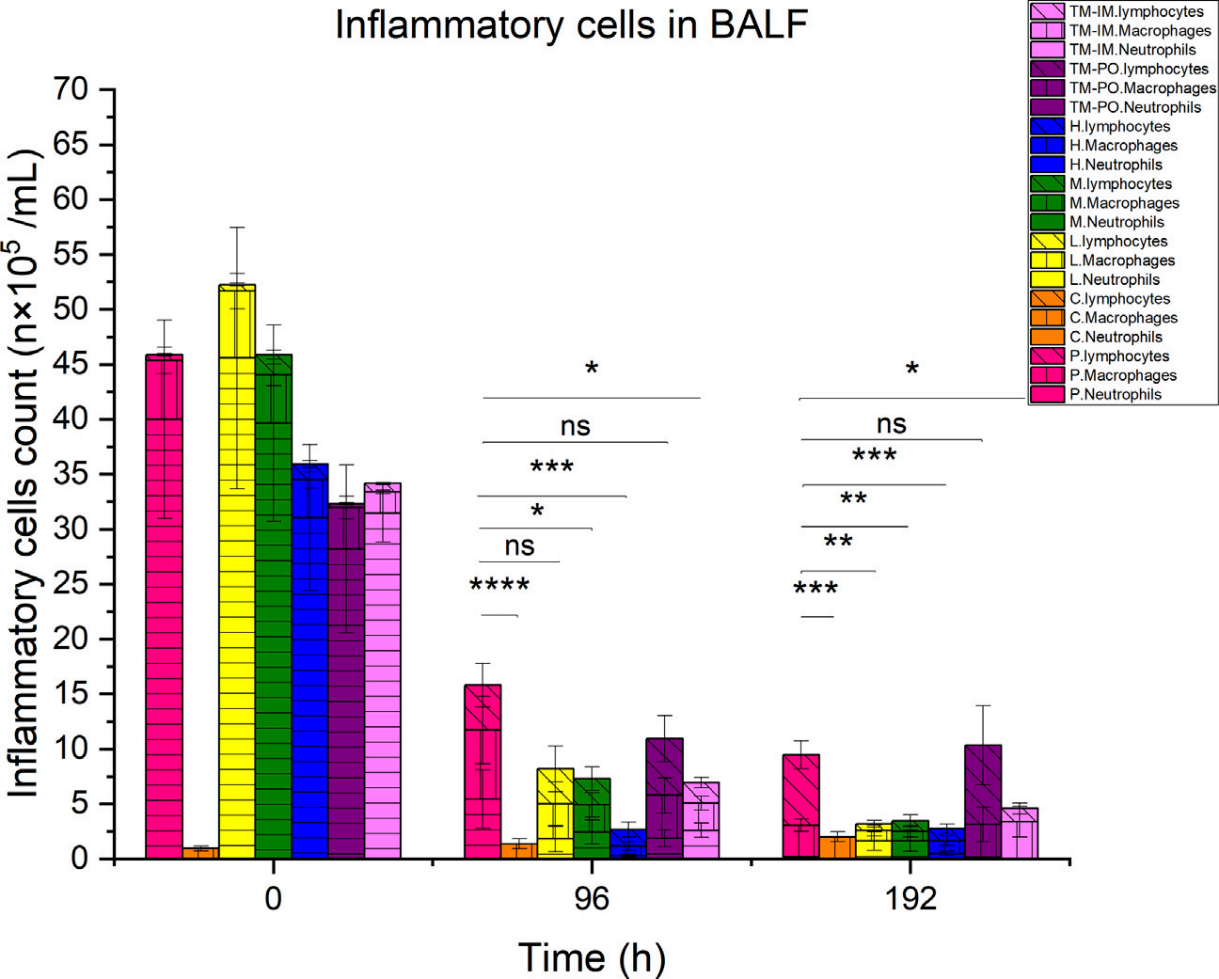
Differential inflammatory cell counts in BALF of experimental mice. Pink, positive control; orange, control. Yellow, green, and blue represent the low, medium, and high dose groups of compound16C, respectively; purple represents the tiamulin oral administered group, and lilac represents the intramuscular administered group. Horizontal, vertical, and oblique stripes represent neutrophils, macrophages, and lymphocytes, respectively. n = 6 at each time point. *P< 0.05; **P< 0.01; ***P< 0.001; ****P< 0.0001. n = 6 at each time point.

Our mouse infection model was also utilized to examine the accumulation of inflammatory cells in the BALF of the mice during the treatment process. Infection with *M. pneumoniae* resulted in significant increases in total BALF cells compared to non-infected controls, which were primarily neutrophils. Following 4 days of continuous treatment, the total number of inflammatory cells decreased, and these levels were significant for compound 16C medium (P< 0.05) and high (P< 0.001) dosages compared with the positive controls. Although the number of cells in low-dose and tiamulin orally administered groups was lower than for the positive controls, the difference was not statistically significant. Similar to the medium dose group of compound 16C, the number of inflammatory cells in the intramuscular administration of the tiamulin group was also significantly lower than that in the positive control group. However, we did find a significant decrease in neutrophils while macrophage and lymphocyte levels increased. Four days following treatment cessation, each group’s cell counts did not recover did not recover. The number of cells in the 20, 40, and 80 mg/kg dose groups for compound 16C and tiamulin intramuscular administered group was lower than that of the positive control and the tiamulin oral administered groups but similar to that of the control group (P> 0.05). Interestingly, the cell community was composed of macrophages and lymphocytes at this time, and neutrophils were rare (Figure 5).

### Assays of cytokines/chemokines in the BALF of experimental mice

**FIGURE 6:**
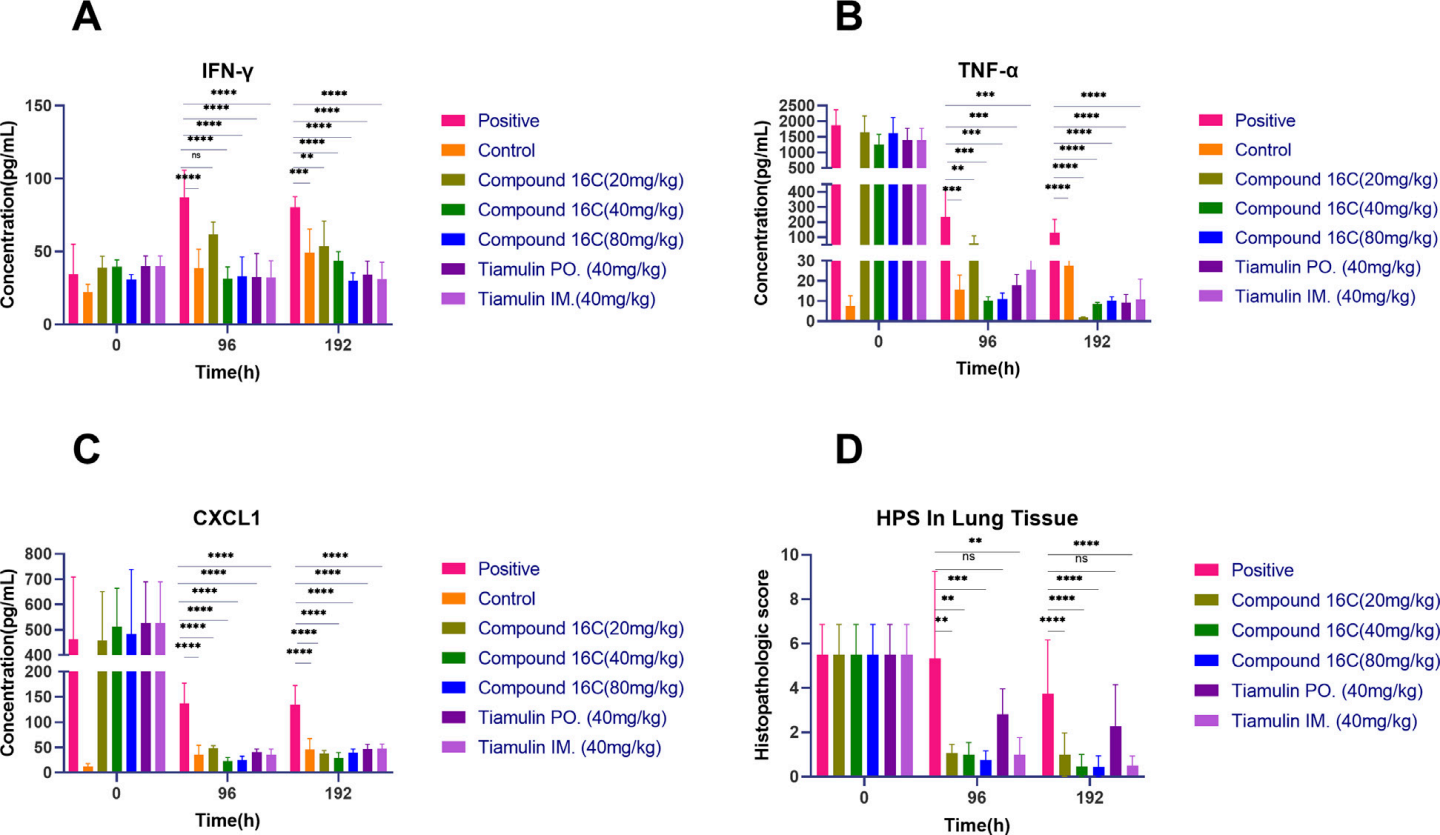
BALF cytokines (n = 6) and Histopathologic scores (HPS) in experimental mice in BALF at the indicated times, cytokines levels in BALF at different time points **(A–C)**; HPS in lung tissue of mice **(D)** (0 h: n = 8, 96 h: n = 6, 192 h: n = 15). *P< 0.05; **P< 0.01; ***P< 0.001; ****P< 0.0001.

We also examined the cytokine content of the BALF we used for the above experiments. At 96 h, all drug-treated mice displayed significant decreases (*p* < 0.01) in the inflammatory markers CXCL1 and TNF-α compared to the positive infection controls, and this was dose-dependent. Four days after the termination of treatment, these biomarkers were still significantly lower than those of the positive control group (Figures 6B, C). However, IFN-γ level increased for the positive controls over time, and drug intervention could prevent these increases. In particular, IFN-γ levels in the positive control group was significantly higher than that in the other groups at 96 h and 192 h (Figure 6A). Interestingly, CXCL1, TNF-α and IFN-γ levels for control mice were elevated in the low level. It was most likely the result of minor inflammation caused by dust in the bedding material and could not be avoided entirely (Figures 6A–C).

### Histopathologic score (HPS) of lung tissue

**FIGURE 7:**
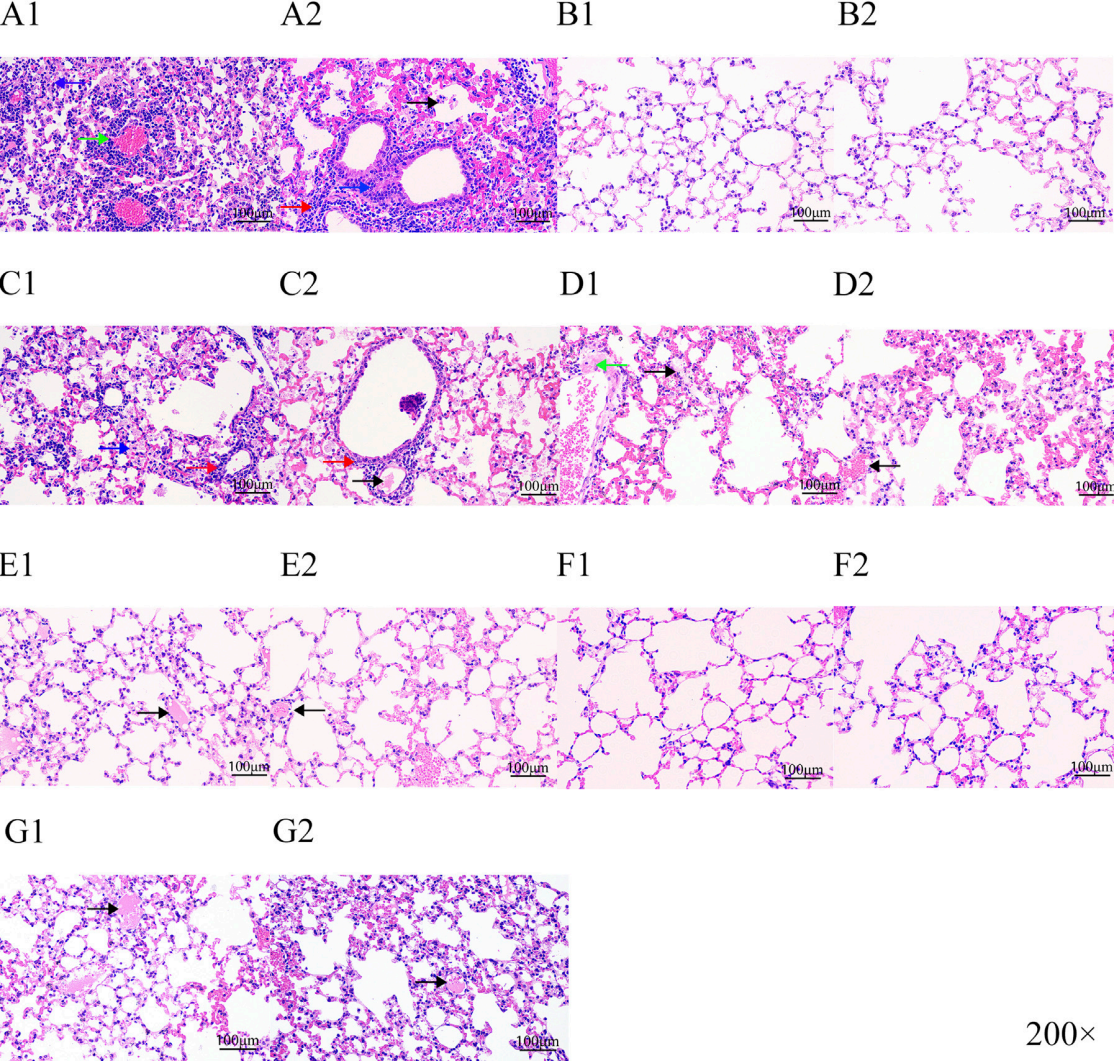
H&E staining of lung tissue sections from experimental mice. Letters represent experimental groupings, and numbers 1 and 2 represent 96 and 192 h, respectively. Red arrow, peribronchiolar/bronchial infiltrates; black arrow, bronchiolar/bronchial luminal exudate; green arrow, perivascular infiltrate; blue arrow, parenchymal pneumoniae. Positive control **(A1-A2)**; control **(B1-B2)**; tiamulin-PO treated **(C1-C2)**; low-dose (20 mg/kg) compound16C **(D1-D2)**; middle-dose (40 mg/kg) compound 16C **(E1-E2)**; high-dose (80 mg/kg) compound 16C **(F1-F2)**; tiamulin-IM treated **(G1-G2)**.

Our experimental animals were also examined for lung lesions following infection and treatment (Figure 7). The normal bronchoalveolar arrangements without any lesions and inflammation were apparent in the lung tissues of control mice. However, *M. pneumoniae-*challenged animals without treatment displayed peribronchiolar/bronchial inflammatory cell infiltrates (red arrow), bronchiolar/bronchial luminal exudate (black arrow), perivascular infiltrate (green arrow) and parenchymal pneumoniae (blue arrow). Through drug intervention, lung lesions were alleviated at 96 h and 192 h after the beginning of drug administration compared with the infected control group. Compound 16C treatment reduced these symptoms and the HPS values at 96 h and 192 h (20 mg/kg: 1.08, 1; 40 mg/kg: 1, 0.46; 80 mg/kg: 0.75, 0.43) of these mice were significantly (20 mg/kg: P< 0.01, P< 0.0001; 40 mg/kg: P< 0.01, P< 0.0001; 80 mg/kg: P< 0.001, P< 0.0001) reduced (Figure 6D). For tiamulin, oral and intramuscular administration showed different therapeutic effects. Although there was no significant difference in HPS value between the drug treatment groups at 96h, the HPS value in the oral tiamulin group was higher than that in the intramuscular tiamulin group. The mean value of HPS in the compound 16C medium dose group was the same as that in the intramuscular tiamulin group (HPS = 1). After 4 days of treatment interruption, the lesions in each group did not aggravate, and the HPS values in the compound 16C treatment groups and the tiamulin-IM group were significantly lower than those in the positive control group and the tiamulin-PO group. Intramuscular compound 16C and tiamulin displayed a more robust response and recovery rate at the middle and high dosage levels than oral tiamulin in these experimental infections (Table 2).

**TABLE 2:** Response and recovery rates of compound 16C and tiamulin in *Mycoplasma pneumoniae-*infected mice (n = 13–15).

	20 mg/kg	40 mg/kg	80 mg/kg	Tiamulin-PO	Tiamulin-IM	Positive
Response (%)	93.33	100	100	86.67	100	46.67
Recovery (%)	20.00	35.7	50	6.67	35.7	0.00

## Discussion

This study evaluated the antibacterial activities of the pleuromutilin derivative compound 16C against *M. pneumoniae* both *in vitro* and *in vivo*. During the pre-screening process, compound 16C possessed a low MIC and excellent *in vitro* antibacterial activity against *M. pneumoniae*, indicating a potential as a new generation of anti-mycoplasma agent. Previous studies also evaluated cell toxicity, and compound 16C displayed only slight influences on the viability of RAW 264.7 and A549 cell lines at the concentration of 8 μg/mL compared with the control drugs. However, the bronchial epithelial cell line 16HBE was slightly more affected but overall, these results indicated that compound 16C was most likely a low or non-toxic compound [30]. We verified this in the current study and found low oral acute toxicity in mice (LD_50_ > 5,000 mg/kg). This level is consistent with other pleuromutilin derivatives, including ATTM (LD_50_ 2,304.4 mg/kg), py-mulin (LD_50_ 2,973 and 3,891 mg/kg for female and male mice, respectively), and Z33 and amphenmulin (LD_50_ > 5,000 mg/kg (Fan et al., 2022; Hu et al., 2022; Zhang et al., 2015; Zuo et al., 2020). However, some compounds have low oral bioavailability and low absorption (Shang et al., 2014; Zuo et al., 2020), which may create the illusion of a lack of toxicity. Therefore, we also assessed the acute toxicity effects of intramuscular administration in mice. The results demonstrated that a single oral or intramuscular administration of 5,000 mg/kg of compound 16C did not induce death, indicating the LD_50_ for both oral and intramuscular administration at > 5,000 mg/kg and that the dosage set for administration in our subsequent experimental protocol was safe. Further studies are needed to evaluate the sub-chronic toxicity of this compound in mice. However, tiamulin showed more potent acute oral toxicity, with LD_50_ of 650 mg/kg in female and 770 mg/kg in male mice, respectively; this substance was more toxic to mice when administered intravenously (50 mg/kg) (EMA, 1999). The hemolysis test evaluates the toxicity of compounds to erythrocytes, and our results showed high hemolysis at high concentrations of the injection solution. This suggests that the injections used in this study have a high hemolysis risk and are unsuitable for intravenous administration; fortunately, we treated MP-infected mice by intramuscular injection, which did not cause symptoms in the mice. Changing the route of administration could avoid the risk of hemolysis. On the other hand, the blank formulation control group showed the same trend as the compound 16C injection group. It suggests that the solvent, such as tween 80 in the formulation, is a significant factor in causing hemolysis (Sæbø et al., 2023). The effect of compound 16C molecule on erythrocytes is still being determined due to solubility limitations, and further experiments are still needed. The results of the cytotoxicity assessment by Chai et al. and the acute toxicity assay in the present study showed a non-toxic propensity of compound 16C (Chai et al., 2022). However, there are still limitations, and we have not thoroughly investigated the effects of the compounds on the organisms, such as the hepatotoxicity, nephrotoxicity and subchronic toxicity of compound 16C. This is necessary to ensure the clinical safety of the drug. Our study aimed to determine the anti-mycoplasma activity of this compound, so only preliminary studies on toxicity were conducted. Systematic studies will be needed to ensure the safety of the compound in the future.

The low water solubility of the prototype derivative (Shang et al., 2014) and the drug’s hepatoenteric circulation may contribute to the low oral bioavailability. Therefore, we examined the pharmacokinetic profiles of intramuscular and intravenous administration routes. Since the software can only obtain AUC_0-∞_ under the two-compartment model, we also analyzed the concentration data using the non-compartment model and output additional AUC_0-t_ and AUC_0-∞_, and calculated their ratios, which were 97.32% ± 2.89% and 99.20% ± 0.38% in the intramuscular and intravenous groups, respectively, which indicates that the analysis results are reliable (Supplementary Material). After intramuscular injection, the plasma concentration of the drug reached the peak rapidly, and its bioavailability was significantly higher than that of a similar compound amphenmulin in mice (IP: 20.71%, PO: 13.65%) and chickens (IM: 52.14%, PO: 5.88%)) (Wang Y. et al., 2024; Zuo et al., 2020). The bioavailability was similar to that of ATTM in broilers (IM: 75.29%, PO: 72.03%) and that of tiamulin in piglets (IM:73.51% or 75.73%) (Shang et al., 2018; Xianhui, 2010) but lower than that of valnemulin in broilers (IM: 88.81%) (WANG et al., 2011). Consistent with previous studies, the plasma concentration of compound 16C decreased rapidly within a short period after intravenous administration (Jin et al., 2019; Zuo et al., 2021). Despite the advantage of its high bioavailability, compound 16C showed a lower AUC_0-∞_ than amphenmulin at the same dose, with an AUC_0-∞_ of 1.56 h μg/mL for intravenous compound 16C versus 12.23 h μg/mL for amphenmulin, and an AUC_0-∞_ of 1.07 h μg/mL for intramuscular compound 16C, also lower than that for oral (1.67 h μg/mL) and intraperitoneal (2.52 h μg/mL) amphenmulin. This dramatically limits the potency of compound 16C *in vivo*. We used a two-compartment model to fit the data and found a high degree of fit (>0.99). Additionally, the low T_1/2ka_ and T_1/2kel_ values, as well as the high Vd (3.55 L/kg) value, indicated that compound 16C was rapidly absorbed, widely distributed, and rapidly eliminated in animals and was most likely the reason for an inability to maintain a high plasma concentration *in vivo* and low AUC_0-∞_ value. The issue that needs to be considered is that the formulation is an essential factor affecting drug metabolism, and it is unreasonable to only consider the drug delivery method without considering the formulation. In order to exclude the influence of formulation, we used the same formulation of dissolved tiamulin in the subsequent pharmacodynamic experiments in addition to oral administration of tiamulin soluble powder aqueous solution, and the same dose and route of administration were used as controls. The short T_1/2kel_ of compound 16C can also be improved by changing the formulation. For example, high-viscosity carriers such as gels and oils can delay the metabolism of substances in the body. A kind of gels for delivery of morphine sulfate consisted of N-O-carboxy-methylchitosan (NOCC) chemically combined with purified chitosan, which significantly increased the AUC_0-∞_ and mean resident time (MRT) of morphine sulfate in beagle dogs compared to saline, while the increase in T_1/2kel_ was not significant (Tasker et al., 1997). In another study, the elimination of doramectin in calves was delayed by using sesame oil containing ethyl oleate as a carrier, and its Tmax was significantly prolonged (Wicks et al., 1993). Chemical modifications aimed at enhancing solubility, developing sustained-release dosage forms, altering the route of administration, and shortening the dosing interval are potential solutions to address these challenges.

Compound 16C possessed robust *in vitro* antibacterial activity, MIC and MBC values were < control antibiotics, and the MIC was < MIC_90_ of lefamulin against *M. pneumoniae* (0.006 μg/mL) (Eraikhuemen et al., 2021). According to the time-kill curve, only compound 16C could reduce levels of the M129 strain to the LOD (2 log_10_ CFU/mL). The antibacterial effect could not positively correlate with dosage when the drug concentration exceeded 8× MIC, indicating a time-dependent growth inhibition rather than dose-dependent. In contrast, control ciprofloxacin effects were positively correlated with dose. Interestingly, except for erythromycin and tetracycline, antibiotics concentrations that resulted in a ≥3 log_10_ CFU/mL reduction in the bactericidal curves were >MBC. This deviation may be attributed to the limited nutrient availability in the test procedure caused by the small volume of liquid (200 μL) in the 96-well plate compared to the 10 mL tubes. However, tiamulin exhibited poor antibacterial activity in the time-kill test, which could be attributed to the development of drug resistance after a long-term culture. This type of effect has been documented for *Mycoplasma gallisepticum* and tiamulin, leading to specific 23S rRNA mutations (Li et al., 2010).

Our study indicated compound 16C represents a new anti-mycoplasmal drug with a high safety profile in acute toxicity studies. We further utilized the mouse lung infection model to evaluate its *in vivo* antibacterial effects. The short half-life and time-kill curves profile of compound 16C suggested that its action is a time-dependent agent. Treatment regimens for time-dependent drugs are most effective using dose-fractionation to achieve the desired antimicrobial activity (Huang et al., 2020). Therefore, we treated the infected mice every 12 h.

This study therefore was a preliminary evaluation of the anti-mycoplasma activity of compound 16C *in vitro* and *in vivo* and we established a representative infection model using mice as experimental animals. *Mycoplasma* species from livestock and poultry cannot stably colonize in mice, and there are few related studies and only one report (Mei et al., 2023). *M. pneumoniae* is easy to colonize in the lungs of mice, and the *M. pneumoniae* pulmonary infection model in mice is widely used in studies of drugs against *M. pneumoniae* pneumonia (Hardy et al., 2003; Ríos et al., 2004; Salvatore et al., 2009; Tagliabue et al., 2011). Although tiamulin is a veterinary antibiotic, it was approved for treating animal *mycoplasma* diseases (EMA, 1999), with high anti-mycoplasma activity and low price, so it was used as the control antibiotic in this study. In this trial, tiamulin was administered intramuscularly and orally, the two approved administration routes. In order to exclude the influence of the preparation on the therapeutic effect, the tiamulin-IM group used the same dissolution formula as compound 16C.

We found a notable reduction in bacterial loads within each group except the positive group. Additionally, oral tiamulin’s antimicrobial efficacy was comparable to low-dose compound 16C, but intramuscular tiamulin was comparable to medium-dose compound 16C. High-dose (80 mg/kg) compound 16C treatment achieved 2.68 and 2.65 log_10_ CFU/mL reductions at 96 and 192 h, respectively, but did not achieve mycoplasmacidal activity. In a similar manner, children with MPP required a prolonged period of treatment, and a 14-day course of intravenous infusion of clarithromycin was more effective than erythromycin (Zhang et al., 2021). This study’s short duration of continuous treatment might result in the anti-mycoplasma effect not meeting expectations.

Unlike the *mycoplasma* load index, several cytokines significantly decreased after treatment, and the HPS study also indicated that 20%–50% of the mice were cured (HPS reduced to 0). There was a disassociation between pneumonia severity and microbiologic outcome vs. bacterial load, which is a characteristic of MPP infections. Multiple studies using the antibiotics tigecycline, clarithromycin, and cethromycin (Hardy et al., 2003; Ríos et al., 2004; Salvatore et al., 2009; Tagliabue et al., 2011) in MPP treatment in mice have consistently shown this outcome, with significant reductions in inflammatory biomarkers and no significant changes in microbial load over the same period. Macrolides have immunomodulatory activity independent of antimicrobial properties and can inhibit cytokine release in acute inflammation and reduce neutrophil recruitment (Bulska and Orszulak-Michalak, 2014; Labro, 1998). While pleuromutilins were rarely used in human disease treatment, some studies have demonstrated anti-inflammatory effects. Tiamulin and valnemulin can alleviate inflammation via the MAPK signaling pathway (Xiang et al., 2022; Zhang et al., 2009). Compound 16C is likely to have a similar immunomodulatory activity to reduce the inflammatory injury caused by *mycoplasma* infection. *M. pneumoniae* also encodes various virulence factors (Jiang et al., 2021), and antimicrobials that inhibit protein synthesis can theoretically alter virulent factor synthesis. These antimicrobials possibly act to reduce pulmonary inflammation by interfering with the production of *M. pneumoniae* immunogen, such as virulence factors, and not by reducing mycoplasmal numbers. Together, these immunomodulatory effects and inhibition of virulence factor synthesis may contribute to this disassociation phenomenon.

We used acute infection with *M. pneumoniae*, and this modality results in a Th1-type immune response (Fonseca-Aten et al., 2005; Hardy et al., 2001), so we screened for the presence of the Th1 cytokines IFN-γ, TNFα, functional IL-8 (CXCL1) in BALF following infection. Early in infection, IFN-γ is derived from CD8 T cells and NK cells, and the initial IFN-γ synergizes with IL-12 to differentiate CD4 T cells into Th1 cells (Das et al., 2001; Schmitt et al., 1994; Woolard et al., 2005). TNF-α is primarily produced by macrophages and T cells but can also be produced by NK cells and neutrophils. This process induces the release of NO, prostaglandins, and other inflammatory mediators by acting on TNFR1 and TNFR2 receptors, causing the acute inflammatory response (Zelová and Hošek, 2013). Neutrophil infiltration is one of the characteristics of MPP, and CXCL1 is the major chemotactic factor that recruits neutrophils instead of IL-8 in mice (Antoniak et al., 2021; Chen et al., 2016). Compound 16C attenuated the Th1-type immune response induced by *M. pneumoniae* acute infection. It inhibited the release of IFN-γ, CXCL1, and TNF-α, as confirmed by histopathological results and analysis of BALF inflammatory cell numbers. Our results showed that both tiamulin and compound 16C significantly inhibited inflammatory factors. Neutrophils in BALF significantly decreased with disease outcome, and macrophages and lymphocytes increased, consistent with previous infection studies (Kurai et al., 2013). The response rate and recovery rate of compound 16C in the treatment of MPP in mice were dose-dependent, and the self-limited character of MPP explained the 46.67% response rate in the positive control group (Lieberman et al., 2004).

Compound 16C showed potent *in vitro* activity with lower MIC values and more rapid bactericidal rates (time-kill curves) than tiamulin. However, compound 16C treatment showed similar effects to intramuscular tiamulin *in vivo*. For example, the 40 mg/kg compound 16C group did not differ significantly from the intramuscular tiamulin group in terms of *mycoplasma* load, inflammatory cells, inflammatory factors and HPS. We hypothesized that this phenomenon might be related to the C14 side chain. The substitution of 4-((4-nitrophenyl)acetamido)phenyl for 2-diethylaminoethyl may have led to a closer binding of the molecules to the *mycoplasma* ribosomes and enhanced *in vitro* drug efficacy (Chai et al., 2022). The alteration of the side chain may also affect the absorption, metabolism, distribution and elimination of the drug molecules *in vivo*, thus interfering with *in vivo* efficacy. This study did not compare the *in vivo* efficacy of compound 16C with other types of antimicrobial agents. Referring to previous studies, the *in vivo* anti-mycoplasmal activity of compound 16C at low doses (20 mg/kg, IM) was higher than that of tigecycline (10 mg/kg, SC, qd, 1d, and 5 mg/kg, SC, qd) (Salvatore et al., 2009), clarithromycin (25 mg/kg, SC, qd) (Hardy et al., 2003), and cethromycin (25 mg/kg, SC, qd) (Ríos et al., 2004). However, such a comparison has limitations, and the efficacy of the compounds is affected by the dosing regimen and route of administration. Further studies are needed to investigate the advantages of the compound 16C over other types of antimicrobial agents.

In conclusion, our results demonstrated that compound 16C was effective against *M. pneumoniae*, and *in vitro* anti-mycoplasmal activity exceeded that of tiamulin, erythromycin, tetracycline, and ciprofloxacin. When administered intramuscularly, compound 16C also produced a similar strong pharmacological action compared with tiamulin and significantly reduced mycoplasmal loads and inflammatory cytokine levels in the lungs of infected mice. This thereby reduced inflammatory cell accumulation and lung pathological damage. These data suggest that compound 16C has the potential as a novel anti-mycoplasma drug. The pharmacokinetic profiles also indicated a rapid *in vivo* elimination, but this could be modulated by future slow-release formulations to enhance its therapeutic effect further.

## Data Availability

The original contributions presented in the study are included in the article/Supplementary Material, further inquiries can be directed to the corresponding author.
