# Supplementary material for: The novel pleuromutilin derivative 22–((4-((4-nitrophenyl)acetamido)phenyl)thio)deoxy pleuromutilin possesses robust anti-mycoplasma activity both in vitro and in vivo
Source: Front Pharmacol. 2024 Dec 20;15:1491223. doi: 10.3389/fphar.2024.1491223 (PMC11695783; doi:10.3389/fphar.2024.1491223)
Supplement: Supplementary file 2 [file Table1.docx]

**Table 1.** Pharmacokinetic parameters of compound 16C in mice (n=8)

| Parameter | IM(10mg/kg) | IV(10mg/kg) |
| --- | --- | --- |
| Cmax (µg/mL) | 0.50±0.11 | - |
| C0 (µg/mL) | - | 5.58±1.25 |
| Tmax (h) | 0.24±0.09 | - |
| Ka (1/h) | 14.41±7.76 | - |
| Kel (1/h) | 0.33±0.07 | 0.41±0.14 |
| T_1/2ka_ (h) | 0.06±0.03 | - |
| T_1/2kel_ (h) | 2.20±0.52 | 1.89±0.61 |
| AUC_0-∞_ (h·µg/mL) | 1.07±0.23 | 1.56±0.37 |
| Cl (L/h/kg) | - | 6.73±1.67 |
| Vd (L/kg) | - | 3.55±1.32 |
| F (%) | 71.29±19.34 | - |
