# Supplementary figures and images for: The novel pleuromutilin derivative 22–((4-((4-nitrophenyl)acetamido)phenyl)thio)deoxy pleuromutilin possesses robust anti-mycoplasma activity both in vitro and in vivo
Source: Front Pharmacol. 2024 Dec 20;15:1491223. doi: 10.3389/fphar.2024.1491223 (PMC11695783; doi:10.3389/fphar.2024.1491223)

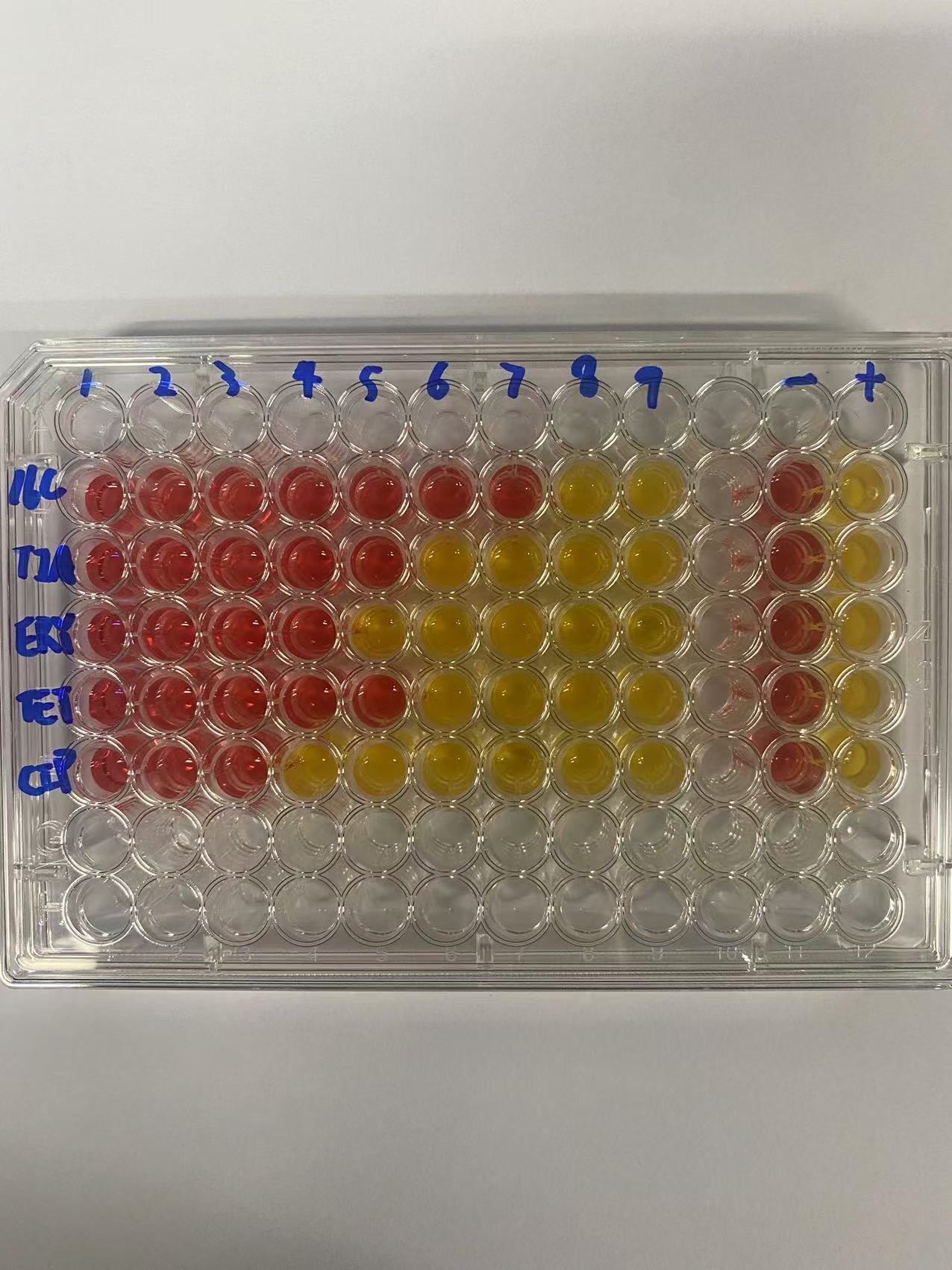

Supplement: Supplementary file 5 [file Image1.JPEG]
