# Supplementary material for: The novel pleuromutilin derivative 22–((4-((4-nitrophenyl)acetamido)phenyl)thio)deoxy pleuromutilin possesses robust anti-mycoplasma activity both in vitro and in vivo
Source: Front Pharmacol. 2024 Dec 20;15:1491223. doi: 10.3389/fphar.2024.1491223 (PMC11695783; doi:10.3389/fphar.2024.1491223)
Supplement: Supplementary file 6 [file DataSheet9.DOCX]

兽药急性毒性试验（LD_50_测定）指导原则

一、概述

（一）定义与目的

急性毒性试验是指一日内对动物单次或多次给药，连续观察给药后动物产生的毒性反应及死亡情况的试验方法。经口（注射）一次性或24h内多次给予受试物后，在短时间内观察实验动物所产生的毒性反应，引起半数致死的剂量称为半数致死剂量，通常用LD50表示。这是评价兽药急性毒性的常用方法。

为了确保急性毒性试验（LD50测定）结果的真实性、可靠性和可追溯性，根据新兽药研究的规律，结合国内兽药毒理学评价的实际情况制定了本指导原则。

（二）适用范围

本指导原则适用于兽用化学药品、中兽药、消毒剂及饲料药物添加剂的急性毒性作用测定。

二、试验设计

（一）材料与方法

1.实验动物

采用两种性别的初成年小鼠和/或大鼠进行试验。小鼠体重为18～22g，大鼠体重为180～220g。动物购买后环境适应饲养3～5天。

2.受试药物及配制

（1）申报兽药的原料或制剂 应为与其他试验同一批号的产品。

（2）受试物溶液配制 一般采用水或食用植物油（如玉米油、花生油、橄榄油等）配制受试物溶液。可考虑用吐温-80作为助溶剂，或用羧甲基纤维素钠、明胶、淀粉等配制成混悬液，不能作成混悬液时，可制备成其它型式（如糊状物等）。必要时可选用二甲基亚砜溶解受试物，但不能采用具有明显毒性的有机溶剂。如采用未知毒性的溶剂应设对照组观察。

3.受试药物给药方法

按受试药物检测要求经口或注射给药。一般一次性给予受试药物。如估计受试物的毒性很低或溶解度很低，可一日内多次给予，每次间隔2～3h，仍合并为一次剂量计算。若总剂量达到5000mg/kg仍不引起动物死亡时，即停止更多次的给予。

给予受试物体积要求小鼠为0.1～0.2mL/10g体重，大鼠为0.5～1.0 mL/100 g体重。经口给予受试物，动物应隔夜空腹进行（一般禁食8～12h左右，不限制饮水）。

（二）试验步骤

1.预试验

每组设4只实验动物（雌、雄各半），拟定高剂量组剂量，再按3～5的倍数递减设定若干剂量组，依次进行试验，找出4/4致死剂量和0/4致死剂量。

如果高剂量组已达5000mg/kg的剂量，实验动物死亡率为0/4，并且将动物数增至10只（雌、雄各半），并重复两次试验动物均未出现死亡，则可结束整个急性毒性试验，急性毒性试验（LD_50_测定）结论为：受试物LD_50_大于5000mg/kg。

2.正式试验

一般设5～7个剂量组。根据预备试验获得的4/4致死剂量（*b）*和0/4致死剂量（*a）*的比值来确定正式试验组数（*N）*；比值为2～3时，设4～5组；比值为3～10时，设5～7组；比值大于10时，设7组。

按下列公式求得相邻两组剂量比值（*r）*：

*r* = log^-1^$\left(\frac{\log b-\log a}{N-1}\right)$

以预试验获得的4/4致死剂量（*b）*作为正式试验高剂量组（第一组）的剂量，按*r*值等比求得其它各剂量组的剂量。

（1）实验动物称重、标记编号和分组

雄、雌动物分开进行正式试验，每组不小于10只动物。分别对雄、雌动物进行编号标记及称重，按设计试验组数（*N）*对实验动物进行分组。分组采用完全随机法。

（2）受试药物溶液配制

按“1：K系列稀释法”等容量配制各剂量组所需的受试药物溶液，根据设计的试验剂量，按试验动物给予受试药物的体积要求，首先确定最大剂量组（如第一组）所需配制的受试药物溶液浓度*C*_1_以及各剂量组所需受试药物溶液的体积*v*。

受试药物母液应配制的浓度C=*C*_1_，应配制的体积V（mL）按下列公式计算：

*V*=$\frac{v}{1-k}$（其中*K*=$\frac{1}{r}$）

根据母液浓度及体积计算需称取的受试药物量（mg或mL）。

称取受试药物，置烧杯内，加入选定的溶剂溶解或稀释，转入容量瓶内，充分混匀并定容，即获得浓度C=*C*_1_的受试物母液。从中取出供最大剂量组（如第一组）给药用的溶液体积*v*，向原溶液中加入同体积的溶剂，混匀后溶液浓度为*C*_1_×*K*，正好是第二组所要求的剂量浓度*C*_2_；从中取出供第二组给药用的溶液体积*v*，再加入同体积的溶剂，混匀后溶液浓度为*C*_2_×*K*，正好是第三组所要求的剂量浓度*C*_3_。依此类推，配制得到各剂量组所需浓度及体积的受试药物溶液。

（3）给药方法

按实验动物经口（注射）给予受试药物的体积要求，根据各实验动物的体重，计算各实验动物给予的受试药物体积。

按要求保定实验动物进行灌胃或注射给药。如在给药过程中因挤压、呛肺等造成死亡，需及时补足每组的动物数量。

（4）试验观察

实验动物给药后一般观察7天，若给药4天后继续有死亡的，则需观察14天，必要时可延长观察至28天。认真观察实验动物中毒的发生、发展过程(观察项目见6.1)，获取中毒特点及毒作用特征，对死亡动物需进行解剖，观察其中毒病理变化，并作好记录。

（三）数据整理

当雄性和雌性实验动物对受试药物反应差异显著时，应分别对其试验数据进行整理，计算各自的半数致死量（LD_50_）；性别差异不显著，则将雄性和雌性实验动物的试验数据集中整理，统一计算分析。

根据试验记录，整理获得下表中的各项数据。

| 组别 | 动物数（*n*） | 给药剂量  （mg/kg或mL/kg） | 剂量对数  （x） | 死亡动物数  （*r*） | 死亡率  （*p*） | 存活率  （*q*） | *p·q* |
| --- | --- | --- | --- | --- | --- | --- | --- |
|  |  |  |  |  |  |  |  |
| …… |  |  |  |  |  |  |  |

半数致死量（LD_50_）计算方法：

将试验获得的各项数值代入下列公式

LD_50_=lg^-1^$\left[Xm-i\left(\sum p-0.5\right)\right]$

S_x50_=$i\sqrt{\sum\frac{pq}{n}}$

LD_50_的95%可信限= lg^-1^（lgLD_50_±1.96×S_x50_）

式中：

*Xm* —— 最大剂量的对数值；

*i* —— 组距，即相邻两组剂量对数值之差；

*p* —— 各剂量组死亡率（以小数表示）；

*q* —— 各剂量组存活率，*q* =1-*p*；

$\sum p$—— 各剂量组死亡率之和；

*n* —— 各组动物数；

S_x50_ —— lgLD_50_的标准误差

（四）结果评定

根据实验动物给药后中毒症状出现的时间、症状表现、死亡出现时间、尸体解剖病理变化及半数致死量（LD_50_），参照表3-2化学物急性毒性（LD_50_）剂量分级表，对受试药物进行评定。评定内容包括：

受试物主要中毒症状表现；

受试物半数致死量(LD_50_)及其95%可信限（单位mg/kg）；

受试物毒性分级；

（五）注意事项

1.本指导原则仅对寇氏（korbor）法的技术环节及要求进行了规定，采用其它方法进行急性毒性试验需参照国家标准GB 15193.3-2003的相关内容进行。

2.除特别注明外，试验用水为蒸馏水，试剂为分析纯试剂，动物为SPF级实验动物。

3.实验动物饲养环境要求达到SPF级。

三、试验报告

为公正、科学地评价药物的毒性，对试验报告内容做如下要求：

1.试验目的。

2.试验时间与地点。

3.试验设计者、负责人、参加者及电子邮箱。

4.受试药物需注明兽药名称、生产厂家、规格、生产批号及用法与用量。

5.试验动物的品种与品系、体重、日龄或月龄、健康状况、检疫情况等。

6.归纳总结该药物的试验结果，确定受试药物的LD50和并对其急性毒性进行评定等。

7.参考文献。

8.试验数据，应有详细的试验原始记录。原始资料保存处、联系人、电话。

9.试验单位（加盖公章）。

四、附录及说明

附表4-1 啮齿动物中毒表现观察项目

| 器官系统 | 观察及检查项目 | 中毒后一般表现 |
| --- | --- | --- |
| 中枢神经系统及  躯体运动 | 行为 | 改变姿势，叫声异常，不安，呆滞 |
|  | 动作 | 震颤，运动失调，麻痹，惊厥，强直性动作 |
|  | 各种刺激的反映 | 易兴奋，知觉过敏或缺乏知觉 |
|  | 大脑及脊髓反射 | 减弱或消失 |
|  | 肌肉张力 | 强直或弛缓 |
| 自主神经系统 | 瞳孔大小 | 缩小或放大 |
|  | 分泌 | 流涎，流泪 |
| 呼吸系统 | 鼻孔 | 流鼻涕 |
|  | 呼吸性质和速率 | 徐缓，困难，潮式呼吸 |
| 心血管系统 | 心区触诊 | 心动过缓，心律不齐，心跳过强或过弱 |
| 胃肠系统 | 腹形 | 气胀或收缩，腹泻或便秘 |
|  | 粪便硬度和颜色 | 粪便不成形，黑色或灰色 |
| 生殖泌尿系统 | 阴户，乳腺 | 膨胀 |
|  | 阴茎 | 脱垂 |
|  | 会阴部 | 污秽 |
| 皮肤和被毛 | 颜色，张力 | 发红，皱褶，松弛，皮疹 |
|  | 完整性 | 竖毛 |
| 粘膜 | 粘膜 | 流粘液，充血，出血性紫绀，苍白 |
|  | 口腔 | 溃疡 |
| 眼 | 眼睑 | 上睑下垂 |
|  | 眼球 | 眼球突出或震颤 |
|  | 透明度 | 混浊 |
| 其他 | 直肠或皮肤温度 | 降低或升高 |
|  | 一般情况 | 姿势不正常，消瘦 |

附表4-2 化学物急性毒性（LD_50_）剂量分级表

| 级别 | 大鼠口服LD_50_/（mg/kg） | 相当于人的致死量（g/人） |
| --- | --- | --- |
| 极毒 | ＜1 | 0.05 |
| 剧毒 | 1～50 | 0.5 |
| 中等毒 | 51～500 | 5 |
| 低毒 | 501～5000 | 50 |
| 实际无毒 | 5001～15000 | 500 |
| 无毒 | ＞15000 | 2500 |
