# Supplementary material for: The novel pleuromutilin derivative 22–((4-((4-nitrophenyl)acetamido)phenyl)thio)deoxy pleuromutilin possesses robust anti-mycoplasma activity both in vitro and in vivo
Source: Front Pharmacol. 2024 Dec 20;15:1491223. doi: 10.3389/fphar.2024.1491223 (PMC11695783; doi:10.3389/fphar.2024.1491223)
Supplement: Supplementary file 8 [file Table2.docx]

**Table 2.** Response and recovery rates of compound 16C and tiamulin in *M. pneumoniae-*infected mice (n=13-15)

|  | 20mg/kg | 40mg/kg | 80mg/kg | Tiamulin-PO | Tiamulin-IM | Positive |
| --- | --- | --- | --- | --- | --- | --- |
| Response (%) | 93.33 | 100 | 100 | 86.67 | 100 | 46.67 |
| Recovery (%) | 20.00 | 35.7 | 50 | 6.67 | 35.7 | 0.00 |
